# Lung Cancer Gene Signatures and Clinical Perspectives

**DOI:** 10.3390/microarrays2040318

**Published:** 2013-12-13

**Authors:** Ruprecht Kuner

**Affiliations:** 1 Unit Cancer Genome Research, German Cancer Research Center and National Center for Tumor Diseases, Heidelberg 69120, Germany; E-Mail: rkuner@t-online.de; Tel.: +49-6221-56-5958; Fax: +49-6221-56-5382; 2 Translational Lung Research Center Heidelberg (TLRC-H), German Center for Lung Research, Heidelberg 69120, Germany

**Keywords:** lung cancer, NSCLC, biomarker, gene signature, testing

## Abstract

Microarrays have been used for more than two decades in preclinical research. The tumor transcriptional profiles were analyzed to select cancer-associated genes for in-deep functional characterization, to stratify tumor subgroups according to the histopathology or diverse clinical courses, and to assess biological and cellular functions behind these gene sets. In lung cancer—the main type of cancer causing mortality worldwide—biomarker research focuses on different objectives: the early diagnosis of curable tumor diseases, the stratification of patients with prognostic unfavorable operable tumors to assess the need for further therapy regimens, or the selection of patients for the most efficient therapies at early and late stages. In non-small cell lung cancer, gene and miRNA signatures are valuable to differentiate between the two main subtypes’ squamous and non-squamous tumors, a discrimination which has further implications for therapeutic schemes. Further subclassification within adenocarcinoma and squamous cell carcinoma has been done to correlate histopathological phenotype with disease outcome. Those tumor subgroups were assigned by diverse transcriptional patterns including potential biomarkers and therapy targets for future diagnostic and clinical applications. In lung cancer, none of these signatures have entered clinical routine for testing so far. In this review, the status quo of lung cancer gene signatures in preclinical and clinical research will be presented in the context of future clinical perspectives.

## 1. Introduction

The ultimate goal reducing the high mortality rate in lung cancer disease is strongly linked to an increased efficacy of cancer prevention strategies and screening approaches for risk assessment and early detection of lung cancer in a curable stage. Major risk factors for lung cancer onset are smoking and increasing air pollution in metropolitan areas [1]. Despite enhanced prevention campaigns in the last two decades, lung cancer still represents the second most frequent malignancy and the highest cancer-related death rate in western countries. In 2013, about 228,190 new cases and 159,480 related deaths were estimated for cancer in the lung and bronchus in the United States [2]. About 55%–60% of patients are diagnosed at late incurable stages with distant metastases. As a consequence, the five-year survival rate is only 13%–16% for all stages. Non-small cell lung cancer (NSCLC) is the most common bronchial tumor, which is classified into the two major histological subtypes adenocarcinoma and squamous cell carcinoma. Both subtypes strongly differ in DNA copy number, DNA methylation, gene mutations, transcriptome, proteome and putative biomarkers as outlined in the following chapters. The stratification of diverse lung cancer entities based on clinico-histopathology and molecular alterations also determines disease outcome and therapy options. Despite significant progress in the development of novel targeted therapies, the high mortality rate in lung cancer strongly emphasizes the need for efficient lung cancer prevention and screening approaches, and the better stratification of patients who will benefit from a particular therapy regimen. A survey of clinical studies between 2009 and 2012 indicated enlarged activities of biomarker analyses to almost half of all interventional studies [3]. Biomarker-based patient selection for therapy decision clearly increased up from 7.9%–25.8%. The major goal is to identify and validate specific biomarkers or signatures in lung cancer tissues and patient surrogates, that will shift lung cancer diagnosis towards a curable stage, better stratify patients with resectable tumors for the need of adjuvant therapies, and guide clinicians to select the most beneficial therapy regimens for their patients after first diagnosis and disease progression. 

## 2. Risk Assessment and Early Detection of Lung Cancer

In the National Lung Screening Trial, low-dose computed tomography-based lung cancer screening reduced cancer mortality in high-risk individuals [4]. However, CT screening is also accompanied with a high rate of suspicious cases without confirming a malignancy, cancer over-diagnosis and economic challenges [5]. A future diagnostic testing scenario may also include screening approaches for cancer-related nucleic acid, peptide or metabolic molecules. So far, no molecular test for lung cancer diagnosis has been established in routine health care. Serum proteins like CEA, CYFRA 21-1 or MALDI/MS signatures might have been valid to detect lung cancer subtypes, but did not overcome clinical studies [6]. 

An immunobiomarker test (EarlyCDT^®^-Lung) measuring autoantibodies to a panel of seven antigens (p53, NY-ESO-1, CAGE, GBU4-5, SOX2, HuD, and MAGE A4) in serum has been developed [7,8], and is actually evaluated in a phase 2 trial. This commercially available assay objects the early detection of lung cancer in high-risk individuals (long standing smokers) and risk stratification in patients with pulmonary nodules detected by CT scans. Depending on cut-off criteria, sensitivity (49%) and specificity (93%) leads to the diagnosis of one lung cancer patient from seven positive tested individuals [9]. Another *in vitro* diagnostic test assay (Epi proLung BL Reflex Assay, Epigenomics, AG) measures *SHOX2* DNA methylation in bronchial aspirates of patients which are suspected for lung cancer (78% sensitivity, 96% specificity), and is suggested as diagnostic adjunct when cytology results are negative or suspicious [10]. Additionally, a clinical trial is ongoing to test the accuracy of mediastinal staging by *SHOX2* methylation level in transbronchial needle aspiration [11]. Moreover, a 4-gene methylation signature (*p16*, *TERT*, *WT1*, and *RASSF1*) was reported based on 655 bronchial washings to diagnose lung cancer with 82% sensitivity and 91% specificity [12]. However, it remains a challenge to sequentially combine different methods like CT and genomic signatures in a screening approach and to avoid a high number of false positives.

Circulating miRNAs, robustly detected in serum and plasma, are suggested as promising biomarkers in cancer patients. Different abundance of specific miRNAs was detected in serum and plasma of lung cancer patients, which might improve risk group assessment for further CT and invasive diagnostics [13,14,15,16]. One study outlined a weighted linear combination of the expression levels of 34-miRNAs measured in 253 patients separated in a training, validation and an additional clinical validation cohort finally displaying 71% sensitivity and 90% specificity [13]. A 10-miRNA signature developed in a screening and validation study including serum from 620 NSCLC patients and controls proposed a better accuracy (90% sensitivity and 93% specificity) for early lung cancer detection [15]. Of note, expression of a serum miRNA pair (miR-15b and miR-27b) promised a 100% sensitivity and 84% specificity [16]. The increasing number of studies reporting circulating miRNAs as putative biomarkers in cancer patients indicates the great potential in biomarker discovery and translational research. Blood cells were also analyzed for non-invasive biomarkers. For example, a large gene classifier was identified and validated in blood of 233 patients using Illumina microarrays [17]. In contrast to serum and plasma, overall RNA expression will be strongly affected by various compositions of blood cell types. 

Molecular alterations have been associated with the individual risk for lung tissue damage and tumorigenesis. The comparison between lung cancer and benign tissues revealed diverse transcriptional profiles and putative diagnostic biomarkers [18,19,20]. For example, comprehensive meta-analysis of 20 studies comprising over 1100 lung tumor and benign tissues resulted in a robust tumor-associated 15-miRNA signature in NSCLC [20]. miRNA detection methods include both microarray and qPCR technology. Technical studies revealed a higher variation of miRNA quantification between different microarray platforms compared with qPCR and sequencing, and proposed a higher sensitivity and specificity for qPCR-based miRNA expression analysis [21,22]. Recently, an 8-miRNA signature (miR-96, miR-450a, miR-183, miR-9, miR-577, Let-7i, miR-27b miR-34a) was proposed to diagnose NSCLC in minimal biopsy material [23]. In non-tumor lung tissues of 853 lung cancer patients large and consistent gene expression variances caused by smoking were identified by using microarray technology [24]. Furthermore, gene expression changes along the airways were analyzed in order to investigate if easily accessible epithelial cells like in the nose, mouth or main bronchus reflect early oncogenic alteration in lung tissues caused by toxic agents like cigarette smoke [25,26]. Here, a prospective study (DECAMP-1) was started in 2013 aiming at the validation of gene, protein and cytokine signatures identified in bronchial airway and serum of cancer patients [27]. 

Alternative lung specimens like endobronchial epithelial lining fluid, bronchial lavage and sputum are collected for biomarker research. For example, DNA methylation changes, specific gene and miRNA expression signatures were identified in the presence of cancer cells [28,29,30]. However, standardization of sampling procedures for these approaches is much more challenging, because the general health condition and comorbidities strongly affect sample recovery and cellular components.

As non-invasive approach, GC/MS or electronic noses were used to identify diverse signatures of volatile organic compounds (VOCs) in exhaled breath of cancer patients compared to healthy individuals [31,32,33]. Distinct VOC profiles could be assigned to patients suffering from lung cancer. In addition to analytical techniques, the excellent olfactory sense of dogs might contribute to early lung cancer detection [34]. Here, a phase-2 trial was terminated in 2013 because of inconsistent training status of sniffer dogs. Such diagnostic methods have the potential to improve the stratification of high-risk individuals and suspicious cases before invasive diagnostic bronchoscopy. Ongoing validation of diagnostic biomarker profiles and an earlier focus on standardization parameters of the techniques are prerequisites for application into clinical practice.

## 3. Molecular Stratification of Non-Small Cell Lung Cancer Subtypes and Outcome

### 3.1. Molecular Profiling of NSCLC Tumor Subtypes

After lung cancer detection, clinicopathological parameters like tumor histology, staging and localization of metastases determine the disease outcome and current therapeutic interventions [35,36]. The clinical practice guidelines differentiate between small cell lung cancer (SCLC) and NSCLC, and between the major NSCLC subtypes squamous and non-squamous cell carcinoma assessed by standard histopathology. Concerning the status of present blood biomarkers, different serum levels and ratios of ProGRP, CEA, SCC, CA 125, CYFRA 21-1 and NSE have been proposed to distinguish between the two major NSCLC subtypes adenocarcinoma and squamous cell carcinoma, and SCLC, respectively [37]. Here, the precise determination of protein isoform signatures in lung cancer patients may further improve testing accuracy [38]. Recently, Roche Diagnostics launched Elecsys ProGRP test for a more precise diagnosis of SCLC from patients’ serum and plasma. 

The access of resected tumor material enables a comprehensive cancer-cell related diagnostics and molecular profiling. In the past, numerous microarray-based profiles were reported after millennium. Diagnostic and prognostic gene signatures using gene expression microarrays were outlined in Tables A1 and A2. Based on transcriptional profiles lung adenocarcinomas were stratified in molecular subgroups proposing diverse cellular characteristics and prognosis [18,39,40,41,42,43]. Gene expression patterns were associated with diverse adenocarcinoma subtypes named bronchioid, squamoid, and magnoid, dependent on transcriptomic similarities with histologically defined bronchioalveolar carcinoma, squamous cell carcinoma, and large-cell carcinoma [41]. These expression profiles were further investigated across six independent studies and about 1,000 patients [44]. Here, distinct molecular alterations, mutations, copy number variation and methylation could be assigned to these intrinsic subtypes with implications for further therapy modalities. Another study differentiated between two different molecular subtypes with respect to a prognostic 193-gene signature [42]. Recently, architectural classification of invasive pulmonary adenocarcinomas described five predominant patterns and has been shown to be a stage-independent predictor of survival [45,46]. Few molecular markers like TTF-1 have been investigated across IASLC/ATS/ERS adenocarcinoma architectures and were associated with disease recurrence [47,48]. The occurrence of different histological pattern in one tumor impedes the evaluation of the predominant architecture. Moreover, the histopathological AC patterns do not directly correspond with the molecular subtypes mentioned above. However, gene expression analysis of distinct architectural patterns upon tissue microdissection is under way to select for specific signatures and novel targets. 

Similar subtype analyses were done for squamous cell carcinomas of the lung predicting different survival outcomes [19,49,50]. The stratification of four different SCC gene expression subtypes named primitive, classical, secretory and basal generated by a nearest-centroid predictor from microarray data was reproducible across independent microarray and RNA sequencing datasets including about 600 SCCs [50,51]. The primitive subtype was associated with the worst survival outcome. Furthermore, gene expression patterns in distinct molecular subtypes were attributed to the activation status of biological and cellular processes, and oncogenic pathways. A better understanding of the relationship between histopathological diversification of lung tumors, molecular characteristics and disease outcome will contribute to molecular pathology and biomarker development in the future. 

Nowadays, comprehensive genomic and transcriptomic sequencing allows an integrative analysis of gene expression, mutations, copy number variations and DNA methylation to assign more complex signatures in tumor subgroups [44,51]. In lung cancer clinics, invasive tissue sampling challenges the evaluation of tumor histology. Especially in small biopsy samples from advanced tumors, specific biomarkers and signatures would be highly useful to guide tumor stratification and outcome prediction. For example, a histology expression predictor for adenocarcinoma, carcinoid, small cell carcinoma, and squamous cell carcinoma was developed using RT-qPCR in FFPE samples [52]. Direct comparison between molecular predictor and pathologist indicated similar accuracy and precision of the biomarker approach. Several qPCR- and microarray-based studies identified miRNA patterns specific for lung cancer subtypes. For example, stratification of adenocarcinoma and SCC histology was done by a 34-miRNA panel measured in FFPE specimen from 205 male smokers [53]. Moreover, two small miRNA panels measured in FFPE samples and bronchial brushing lung specimens have been reported to discriminate SCLC from NSCLC, and SCC from adenocarcinoma (AUC = 0.94–0.99), respectively [54]. Similar accuracy has been achieved by an overlapping 8-miRNA panel measured in preoperative cytologic samples [55]. Ongoing comprehensive screening for specific gene expression signatures, driver mutations and other genomic alterations are promising to identify biomarkers and targets for therapeutic intervention in distinct tumor architectures.

### 3.2. Gene Signatures Associated with Disease Prognosis and Outcome

In the cited microarray studies above, differences between histological and molecular tumor subgroups were often characterized by large gene signatures, which are helpful for the understanding of tumor progression and differentiation but very challenging for the translation into molecular diagnostics. In addition, the tumor-intrinsic subclasses are not implicitly associated with disease outcome. At early tumor stage, stringent signatures are needed to predict individual relapse risk and survival, and to support further therapy decisions. Several microarray study reports stated diverse prognostic gene signatures in adenocarcinoma [39,56,57,58,59], SCC [49,60], or NSCLC in general [58,61,62,63,64,65,66,67]. Prognostic classifier was usually generated from a single microarray dataset by applying a Cox model (endpoints survival time or relapse time) to a training set, and validated in an independent test set or by leave-one-out-cross-validation. Thus, most of the signatures are linked to the local study protocols including patient cohort selection, starting biomaterial, microarray platform and statistics, variables, which explain the large divergence of contributing genes and the challenge of reproducibility. The accuracy and robustness of few gene signatures were successfully tested across independent microarray datasets and study centers. Based on multi-site microarray profiles from 442 adenocarcinomas, it has been shown that the predictive value of gene signatures can profit from the addition of clinical covariates [56]. The large collection of microarray datasets from lung cancer tissues provides a valuable source for the selection and validation of clinically relevant gene signatures. This is also reflected by numerous meta-analysis studies integrating public microarray data for searching and validating prognostic gene sets [42,68,69,70,71,72,73,74]. For example, a 64-gene signature predicting survival of stage I NSCLC patients was derived from a consensus expression set of 4,905 genes across seven different microarray datasets [72].

Small prognostic and predictive gene sets in early-stage NSCLC were described to be applicable to standardized analytical techniques like qPCR technology [63,75,76,77,78,79,80,81,82]. Chen and colleagues validated a five-gene signature (*DUSP6*, *MMD*, *STAT1*, *ERBB3*, and *LCK*) for relapse-free and overall survival across 271 stage I-III NSCLC tumors [77]. It was proposed to test in prospective, large-scale, multicenter studies if patients with a high-risk gene signature might benefit from adjuvant therapy. Furthermore, a 14-gene quantitative PCR assay on formalin-fixed paraffin-embedded tissue predicting survival in resected stage I adenocarcinoma was developed in a cohort of 361 patients and validated in two independent cohorts comprising nearly 1,439 patients [83]. Of note, this assay was successful in ethnically distinct cohorts including US and Chinese lung cancer patients. 

Moreover, several prognostic miRNA signatures in lung cancer tissues have been described [53,84,85,86]. For example, a five miRNA signature (let-7a, miR-221, miR-137, miR-372, and miR-182*) was generated from fresh frozen tumor tissues of a training set (*n =* 56) and tested in two independent patient cohorts (*n =* 118) to be associated with survival and cancer relapse in NSCLC patients [86]. Similarly, Landis and colleagues reported another five miRNA signature (miR-25, miR-34c-5p, miR-191, let-7e, and miR-34a) measured in FFPE tissues predicting survival (*p =* 0.017) in squamous cell carcinoma patients [53]. Two further prognostic miRNA signatures were proposed after microarray profiling of tissues from 527 stage I NSCLC patients dependent on the inclusion of AC and SCC subtypes [84]. As outline for gene signatures above, diverse miRNA signatures are likely reasoned by differences in the selected patient cohorts, biomaterials, platform technologies and statistics. Of note, five frequently reported miRNAs (miR-21, miR-29b, miR-34a/b/c, miR-155 and let-7a) could not be confirmed as prognostic or predictive biomarker in a large cohort (IALT trial) including 639 patients with resectable NSCLC receiving adjuvant chemotherapy [87]. A future diagnostic test based on tissue sections has to consider feasible tissue repository in the clinics, and valid standards to evaluate biomarker molecule quality and tumor cell content.

Distinct circulating miRNA signatures were detected in serum or plasma from early-stage NSCLC patients and associated with recurrence risk and survival [88,89,90]. For example, a signature of four ‘high-risk’ serum miRNAs (miR-486, miR-1, miR-499, miR-30d) was reported to predict overall survival in NSCLC patients (*n =* 303) after surgery and adjuvant chemotherapy [88]. For advanced NSCLC, a combined 17-miRNA signature was able to calculate a 2.5-fold increased risk of death between low- and high-risk score patients [91]. The usage of specific blood biomarkers for risk assessment would facilitate diagnostics, especially for inoperable NSCLC patients where the access of representative tumor tissue is difficult. 

## 4. Predictive Biomarkers for Lung Cancer Therapies

### 4.1. Prognostic and Predictive Biomarkers for Systemic Therapies

Adjuvant therapies are recommended for patients with operable lung cancer dependent on tumor staging. However, the stratification of those patients is still not accurate with regard to the relapse risk and individual response. In other words, no clinical parameter or biomarker is available to predict complete cure after removal of early-stage lung tumor, or to calculate effectiveness of adjuvant chemotherapy in preventing disease relapse. Numerous prognostic signatures in tumor tissues or patient surrogates of early disease patients were generated to potentially improve therapy management if adapted to clinical routine. It has been shown that single biomarkers often failed to efficiently predict therapy response. The well-studied DNA repair genes *ERCC1* and *RRM1* as predictive biomarkers for chemotherapy did not enter clinical routine [92]. Weakness in the performance of detection methods and the intratumoral heterogeneity of biomarkers limited the value of single biomarkers like ERCC1 [93,94]. Cross-validation analysis of Mucin by immunohistochemistry staining in 780 patients indicated that the biomarker was not predictive for overall survival after chemotherapy [95]. Similarly, the presence of *KRAS* mutations was not recommended to select patient for adjuvant chemotherapy [96]. In advanced NSCLC patients, the immunohistochemical status of beta-3 tubulin was not predictive for the benefit of ixabepilone- or paclitaxel-containing regimens reported in a phase II study [97]. In SCLC patients, a combination of serum biomarkers like nucleosomes, NSE, ProGRP and CYFRA 21-1 achieved up to 47% sensitivity at 95% specificity to predict insufficient response to first-line chemotherapy [98]. 

Of note, several prognostic signatures reviewed in the paragraph before were addressed to the need of adjuvant chemotherapy in early-stage lung cancer patients. For example, a 15-gene signature in tumor tissues from stage IB and II NSCLC patients was reported to predict the benefit from adjuvant chemotherapy [66]. An ongoing NCI observation study is recorded to further validate this signature in FFPE specimen by using quantitative nuclease protection and NanoString assays. Moreover, a 12-gene signature predicting the benefit from adjuvant chemotherapy with cisplatin/ vinorelbine was identified by integrative analysis of genetic aberration, genome-wide RNAi data, and mRNA expression data, and successfully validated in two independent datasets [99]. Both retrospective studies integrated published microarray data in order to validate their predictive gene signatures. The variable validation success rates might be reasoned by different microarray platforms, gene probe qualities, and heterogeneous patient cohorts.

Biomarker studies accompanying radiotherapy or immunotherapy are rare. Several studies suggested putative biomarkers to predict the response or toxicity upon radiotherapy [100,101,102,103]. For example, a gene expression classifier was calculated to predict radiosensitivity by comparing microarray expression profiles of the NCI 60 cell line panel and clonogenic survival assay outcome after 2 Gy of radiation [103]. In NSCLC patients, a blood biomarker panel (CRP, LDH, Osteopontin, CA-9 IL-6, IL-8, CEA, CYFRA 21-1, and α-2M) has been successfully tested to predict survival after (chemo-) radiotherapy [104], and a prospective clinical trial has been currently started to correlate blood biomarkers with overall survival. Similarly, a decrease of serum ProGRP has been associated with response to chemo- and radio-chemotherapy in SCLC [105]. So far, the activation status of oncogenic drivers like EGFR is most conclusive to contribute to radiotherapy efficacy [106]. It has been shown that targeting EGFR pathway increased radiosensitivity of tumor cell [107,108]. In contrast, radiation itself may activate diverse oncogenic pathways and benefit disease relapse [109]. Therefore, it is important to better understand the interaction between radiotherapy and oncogenic signaling in tumor cells. Based on phase II trials including melanoma and NSCLC patients treated with immunotherapeutic recombinant MAGE-A3 protein, an 84-gene gene signature was associated with clinical response [110]. The further validation of this gene signature was announced for two phase III trials. Diverse immune signatures were compared and further dissected for their contribution of the tumor genome, host genetic background and environmental factors [111,112]. The increasing number of clinical trials focusing on immunotherapies may strongly benefit from valid predictive biomarkers. 

### 4.2. Prognostic and Predictive Biomarkers for Targeted Therapies

Substantial progress has been achieved in the field of targeted therapies for lung cancer. At advanced inoperable tumor stage, molecular pathology plays an increasing role for tumor characterization, target identification and individualized therapy options. The era of high-throughput tumor genome sequencing and personalized medicine enables a further classification into molecular subtypes based on activated, therapeutically targetable oncogenes. So far, more than 50% of adenocarcinoma and squamous cell carcinoma can be characterized by mutations, fusion genes or amplifications leading to driver activation with potentially effective targeted drugs [113]. Tumor histology guides driver mutation testing and the ability of targeted therapy approaches. For lung adenocarcinoma, *EGFR* mutation and *ALK* rearrangement testing is recommended, *KRAS* mutation testing is suggested by the NCCN guidelines [35]. The biomarker testing is further specified by frequent mutations and reliable analytical techniques [114]. Tumors with *EGFR* mutations preferentially respond to EGFR tyrosine kinase inhibitors (TKIs), tumors with *ALK* rearrangements are associated with response to crizotinib [115,116]. The value of the most frequently mutated gene *KRAS* as predictive biomarker for EGFR-TKI insensitivity is controversially discussed [117,118]. Further targeted drugs are investigated in clinical trials for molecular subtypes harboring *BRAF*, *PIK3CA* or *HER2* mutations, *ROS1* or *RET* rearrangements, or *c-MET* amplification [113]. In about 35% of squamous cell carcinoma, aberrant *FGFR1*, *PDGFRA*, *AKT1* or *DDR2* are putative drug targets for individualized therapy schemes. 

In a simplified diagnostic scenario, genomic alterations in lung cancer are tested for relevant drivers in order to apply suitable drugs. Back to reality, the assessment of an individual drug scheme is impeded by limited predictive value of present biomarkers, less robust testing techniques, pressure of therapy timing, and subsequently initial and acquired resistance. For example, a significant number of EGFR wild-type tested lung cancer patients respond to EGFR-TKIs [119]. In contrast, 30%–40% of patients with EGFR-mutated tumors do not respond to this therapy, and most of the responders develop resistance after few months [35]. Based on specific peaks in mass spectrometry, a commercial serum/plasma-based assay (VeriStrat^®^) was developed to predict response to EGFR TKI therapy, and retrospectively tested in 441 patients of the BR.21 phase III trial comparing outcome of erlotinib *versus* placebo treatment [120]. In this study the test was prognostic for progression-free and overall survival, but was not able to predict for differential survival benefit from erlotinib. The stratification of patients for EGFR-TKI drug sensitivity depends on the mutation type of the target itself, the activation status of EGFR downstream actors and potential bypass signaling [121]. A better understanding of the acquired resistance mechanism can disclose novel therapy options using combinatorial treatments to prevent bypass signaling, or to assess a suitable therapy after relapse against the novel acquired molecular subtype [122]. Thus, additional predictive biomarkers are urgently needed to improve patient stratification and to suggest targets for mono- or combinatorial therapies for the primary tumor and after disease relapse. 

Microarrays have been used to identify gene signatures associated with driver mutations and response to targeted therapies [123,124,125,126]. A 76-gene signature associated with epithelial-mesenchymal transition was generated from gene expression profiles of cell lines and tissues of NSCLC patients, and proposed to predict resistance to EGFR and PI3K inhibitors [124]. Recently, a 47-gene signature associated with sorafenib sensitivity was retrospectively analyzed based on the BATTLE trial [123]. The signature was reported to serve as additional biomarker for the definition of a subgroup of patients with tumors wild-type for EGFR that may benefit from sorafenib treatment. Furthermore, a large gene set derived from microarrays well stratified lung adenocarcinomas in one *ALK*-mutated and two *EGFR*/*KRAS*/*ALK*-mutation negative subgroups [125]. Based on distinct profiles, novel target candidates have been identified in patients of a triple-negative subgroup with worse prognosis. Large comprehensive genomic studies in lung cancer are ongoing to precisely define clinically relevant tumor subtypes by combining histopathology, mutation status, DNA copy number variation, gene and protein expression, and disease outcome. Future lung cancer diagnosis and therapy will benefit from a continuous histological and molecular characterization of the tumor, and its diversity and mutability during an individual disease course, to be one step ahead of the beast. 

## 5. Conclusions and Outlook

In the last years, the number of clinical trials accompanied by biomarker studies has been continuously increasing [3]. The implementation of novel therapies more and more depend on a parallel development of biomarkers for patient stratification. So far, the translation of promising findings from biomarker research studies into valid test assays is an exceptional case for lung cancer. None of the aforementioned diagnostic or prognostic biomarkers and signatures is implemented in actual lung cancer clinical practice guidelines [127]. For advanced lung cancer, immunohistochemistry staining of protein markers can help to assess tumor histology in small biopsies and limited biomaterial [35]. In the case of non-squamous NSCLC *EGFR* and *ALK* testing are recommended by the NCCN guidelines to stratify patients for targeted therapy approaches, and hopefully represent the starting point for a wide range of targeted therapy options in future. 

The progress in chip-based molecular stratification of breast cancer patients for therapeutic intervention indicates the potential of molecular diagnostics for cancer patient care [128,129,130]. An evaluation of six different genomic tests also emphasizes the need of large prospective randomized trial and the potential benefit of integrated clinicopathological factors [130]. The controversial debates and the reservation against genomic tests may also reflect social-economic challenges and competition with well-established clinicopathological standards. In the context of intratumoral heterogeneity and clonal selection throughout disease course, it is very likely that a combination of molecular biomarkers and clinicopathological factors can increase the power of diagnostic tests and therapy decision. 

The knowledge about histopathological and molecular subtypes cleared the way for genomic testing of specific drivers beneficial for a small subset of affected lung cancer patients. In contrast, the statistical requirements for diagnostic and prognostic molecular biomarkers in most of the studies included both a high sensitivity and specificity in an epidemiologically or clinically defined risk population. Of course, a high specificity is very important to avoid a large number of suspicious cases, which would not be manageable in further clinical programs. However, do we really need a high sensitivity? If a biomarker approach for early diagnosis would be able to shift one third of advanced lung cancer diagnoses towards a potentially curable stage, this would have dramatic consequences on therapy options and cancer mortality. The numerous preclinical and clinical studies reporting diagnostic, prognostic, and predictive biomarkers and signatures well reflect the huge activity in the fields of tumor detection, prognostic stratification and molecular subtyping. So far, the clinical utility of many reported microarray-based prognostic gene signatures in lung cancer is questionable [131]. The future translation of genomic tests into clinical practice will strongly depend on the answer to the unambiguous clinical question, the inclusion criteria of the target population, the availability of required biomaterial, robust analytical techniques and standards, and the validation of the biomarker assay in large, prospective, randomized trials.

## Appendix

**Table A1:** Gene expression microarray studies describing gene clusters or gene signatures in lung cancer or NSCLC.

Clinical focus	Tumor Type	Biomaterial	Gene signature	Screening	Validation	Technology	References
Diagnosis	NSCLC	Blood cells	484-feature classifier	*n =* 77	*n =* 156	Illumina microarrays	[17]
Diagnosis	Lung cancer	normal large-airway epithelial cells	80-gene signature	*n =* 77	*n =* 52	Affymetrix microarrays	[26]
Risk, Smoking	Lung cancer	non-tumor lung tissue	599-feature set	*n =* 344	*n =* 509	Affymetrix microarrays	[24]
Prognosis	NSCLC	Tissues	6-gene signature, clinical covariates	*n =* 56	*n =* 59	Affymetrix microarrays	[67]
Prognosis	NSCLC	Tissues	72-gene signature	*n =* 103	*n =* 69	Agilent oligo microarray	[64]
Prognosis	NSCLC	Tissues	17-gene signature	*n =* 91	public dataset; Potti, 2006	Affymetrix microarrays	[62]
Prognosis; Chemotherapy prediction	NSCLC	Tissues	15-gene signature	*n =* 133	public datasets; Potti, 2006; Raponi, 2006; Shedden, 2008; Roepman, 2009; qPCR (*n =* 30)	Affymetrix microarrays	[66]
Prognosis	NSCLC	Tissues	4-gene signature, clinical covariates	*n =* 27	*n =* 138	Affymetrix microarrays	[63]
Prognosis	NSCLC	Tissues	59-gene signature	*n =* 55	public datasets; Bhattacharjee, 2001; Bild, 2006	Affymetrix microarrays	[65]
Prognosis	NSCLC	Tissues	450-gene signature	*n =* 196	public datasets; Bild, 2006; Raponi, 2006; Shedden, 2008; Zhu, 2010; Hou, 2010	Affymetrix microarrays	[61]
Prognosis	NSCLC	Tissues	5-gene signature	*n =* 125	*n =* 60; public datasets; Beer, 2002	cDNA microarray, qPCR arrays	[77]
*In vitro* model; Pathway	Lung cancer	Cell lines, Tissues	Oncogenic pathway signatures	cell line, lung cancer (*n =* 111)	none	Affymetrix microarrays	[132]

**Table A2:** Gene expression microarray studies describing gene clusters or gene signatures in adenocarcinoma or squamous cell carcinoma.

Clinical focus	Tumor Type	Biomaterial	Gene signature	Screening	Validation	Technology	References
AC-Subtypes; Prognosis	Lung cancer–AC	Tissues	Gene clusters	*n =* 139	none	Affymetrix microarrays	[18]
AC-Subtypes; Prognosis	Lung cancer–AC	Tissues	Gene clusters	*n =* 67	none	cDNA microarray	[40]
AC-Subtypes; Prognosis	NSCLC–AC	Tissues	Gene clusters	*n =* 149	none	Agilent oligo microarray	[43]
AC-Subtypes; Prognosis	AC	Tissues	50-gene signature	*n =* 43	*n =* 43	Affymetrix microarrays	[39]
Prognosis	AC	Tissues	54-gene signature	*n =* 48	*n =* 95	Agilent oligo microarray	[57]
Prognosis	AC	Tissues	Gene classifiers; clinical covariates	*n =* 256	*n =* 186	Affymetrix microarrays	[56]
Prognosis	AC	Tissues	82-feature signature	*n =* 60	*n =* 57	Agilent oligo microarray	[59]
Prognosis	AC	Tissues	3-gene signature	*n =* 82	public datasets; Bhattacharjee, 2001; Shedden, 2008	Illumina microarrays	[58]
Integrative analysis	AC	Tissues	None	*n =* 75	none	Affymetrix microarrays	[133]
Integrative analysis	AC	Tissues	EGFR and KRAS associated gene signatures	*n =* 193	none	Affymetrix microarrays	[126]
Genomic subtypes	AC	Tissues	Gene signatures	*n =* 226	none	Affymetrix microarrays	[125]
SCC-Subtypes; Prognosis	NSCLC–SCC	Tissues	Gene clusters	*n =* 48	none	cDNA microarray	[19]
SCC-Subtypes; Prognosis	NSCLC–SCC	Tissues	Gene clusters, 50-gene signature	*n =* 129	*n =* 36	Affymetrix microarrays	[49]
SCC-Subtypes; Prognosis	SCC	Tissues	Subtype predictor	public datasets; Bild, 2006; Lee, 2008; Raponi, 2006; Roepman, 2009	*n =*56	Agilent oligo microarray	[50]
SCC-Subtypes	SCC	Tissues	Subtype predictor	Wilkerson, 2010	*n =* 178	Agilent oligo microarray	[51]
Prognosis	SCC	Tissues	111-gene signature	*n =* 51	*n =* 58	Operon oligo microarray	[60]
